# Supplementary material for: Mapping Loci That Control Tuber and Foliar Symptoms Caused by PVY in Autotetraploid Potato (Solanum tuberosum L.)
Source: G3 (Bethesda). 2017 Sep 12;7(11):3587–95. doi: 10.1534/g3.117.300264 (PMC5675608; doi:10.1534/g3.117.300264)
Supplement: Supplementary file 3 [file 3587FileS1.pptx]

## Slide 1
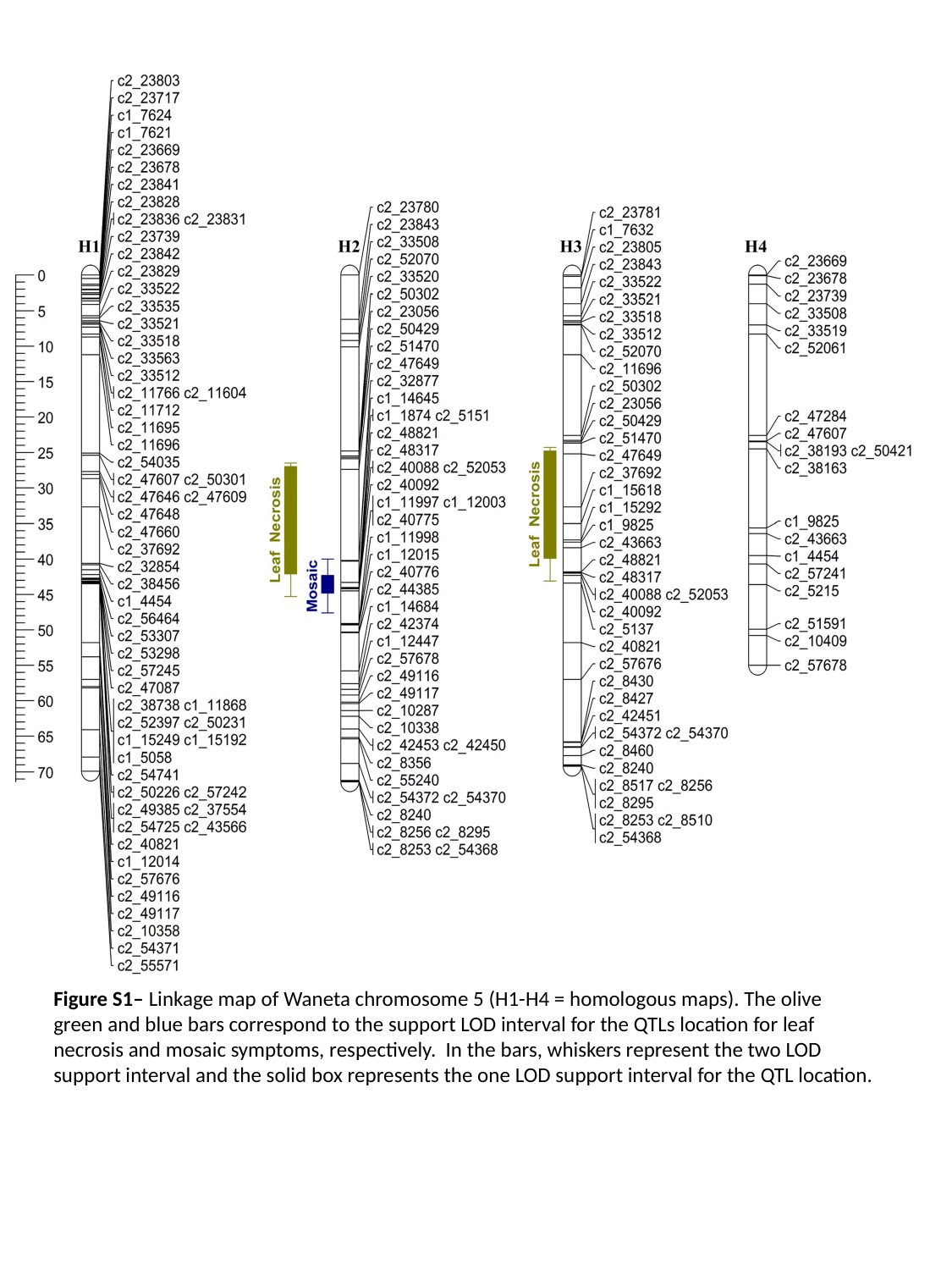

Figure S1– Linkage map of Waneta chromosome 5 (H1-H4 = homologous maps). The olive green and blue bars correspond to the support LOD interval for the QTLs location for leaf necrosis and mosaic symptoms, respectively. In the bars, whiskers represent the two LOD support interval and the solid box represents the one LOD support interval for the QTL location.

## Slide 2
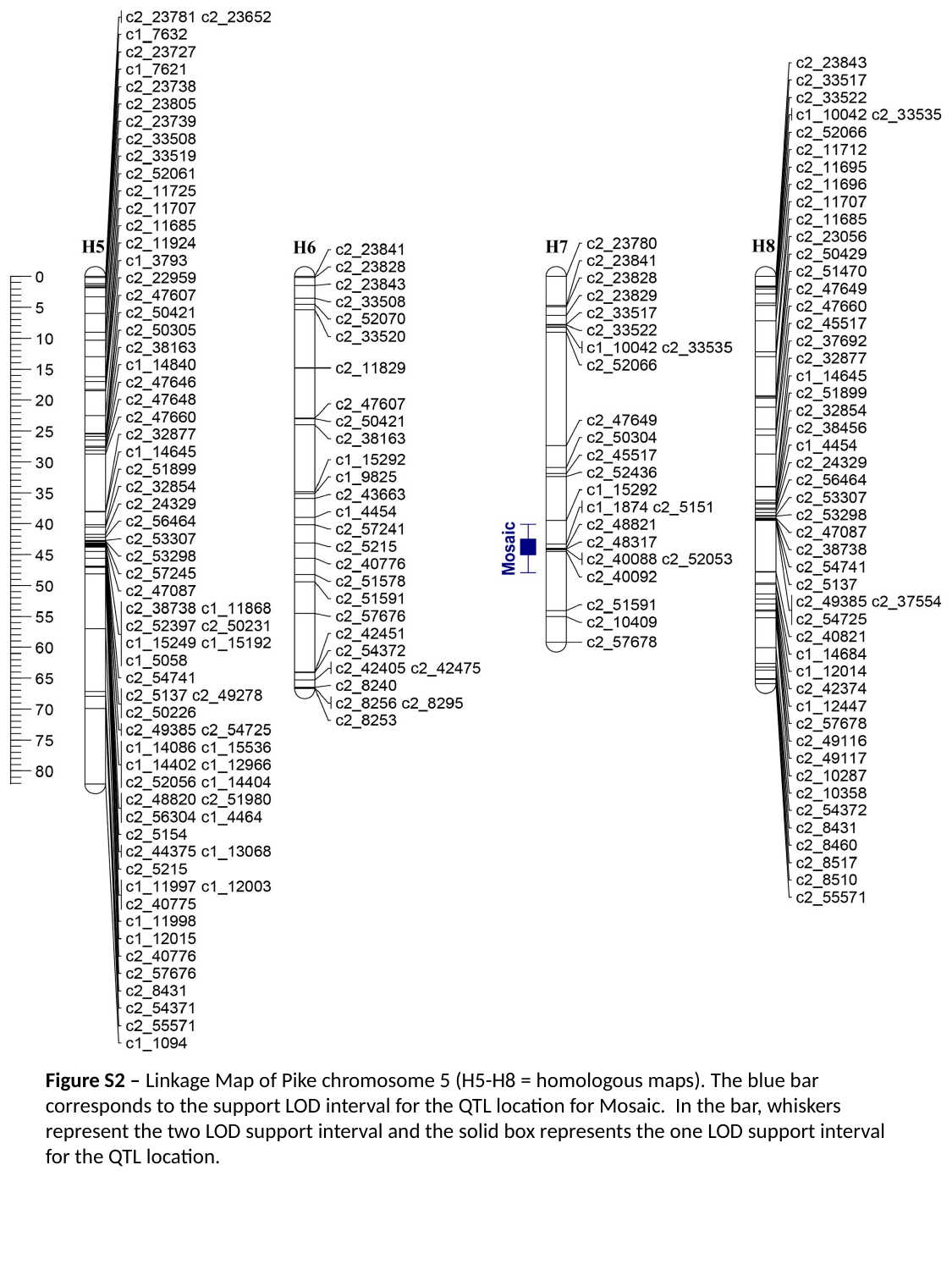

Figure S2 – Linkage Map of Pike chromosome 5 (H5-H8 = homologous maps). The blue bar corresponds to the support LOD interval for the QTL location for Mosaic. In the bar, whiskers represent the two LOD support interval and the solid box represents the one LOD support interval for the QTL location.

## Slide 3
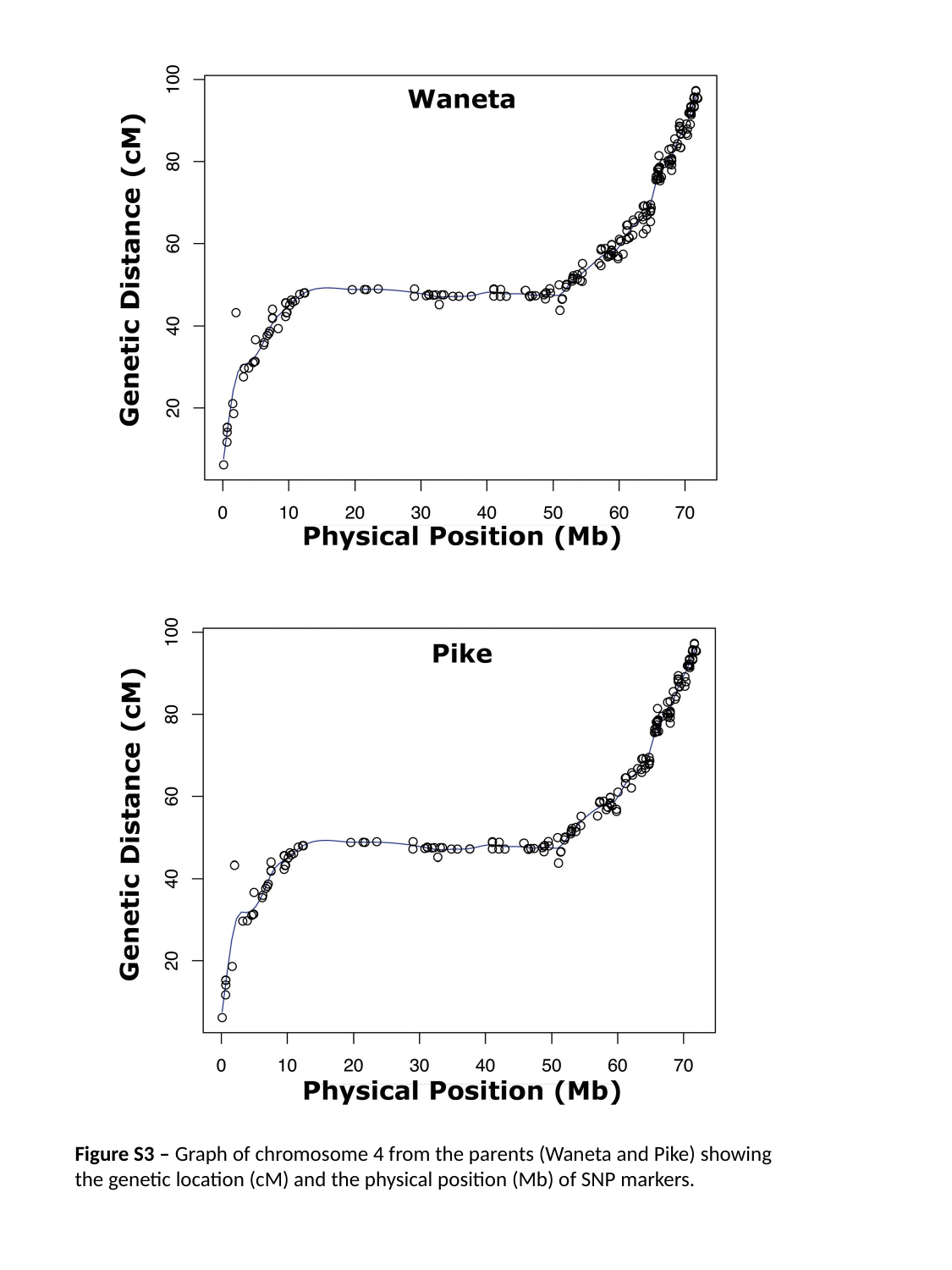

Figure S3 – Graph of chromosome 4 from the parents (Waneta and Pike) showing the genetic location (cM) and the physical position (Mb) of SNP markers.

## Slide 4
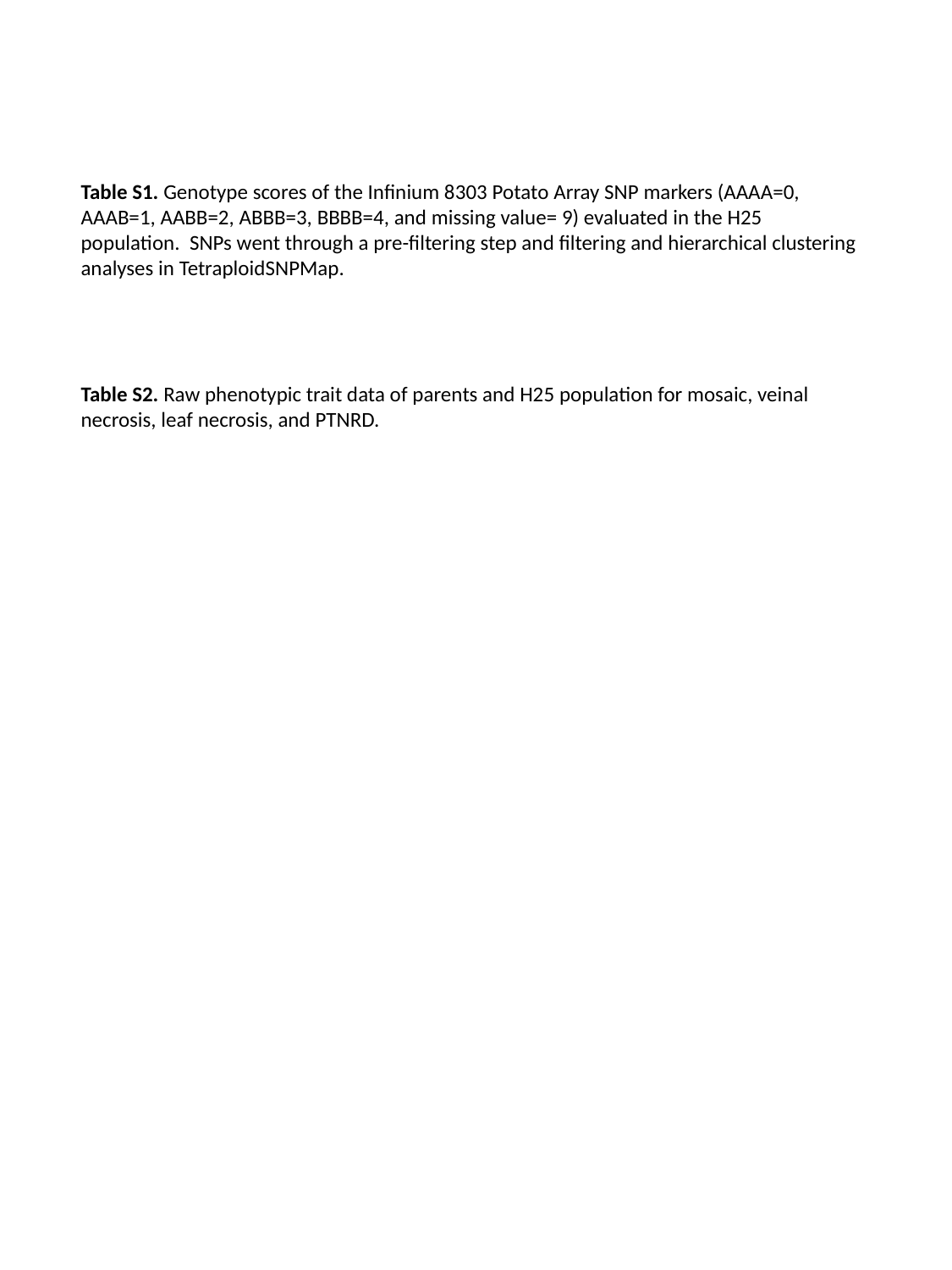

Table S1. Genotype scores of the Infinium 8303 Potato Array SNP markers (AAAA=0, AAAB=1, AABB=2, ABBB=3, BBBB=4, and missing value= 9) evaluated in the H25 population. SNPs went through a pre-filtering step and filtering and hierarchical clustering analyses in TetraploidSNPMap.
Table S2. Raw phenotypic trait data of parents and H25 population for mosaic, veinal necrosis, leaf necrosis, and PTNRD.
| |
| --- |

## Slide 5
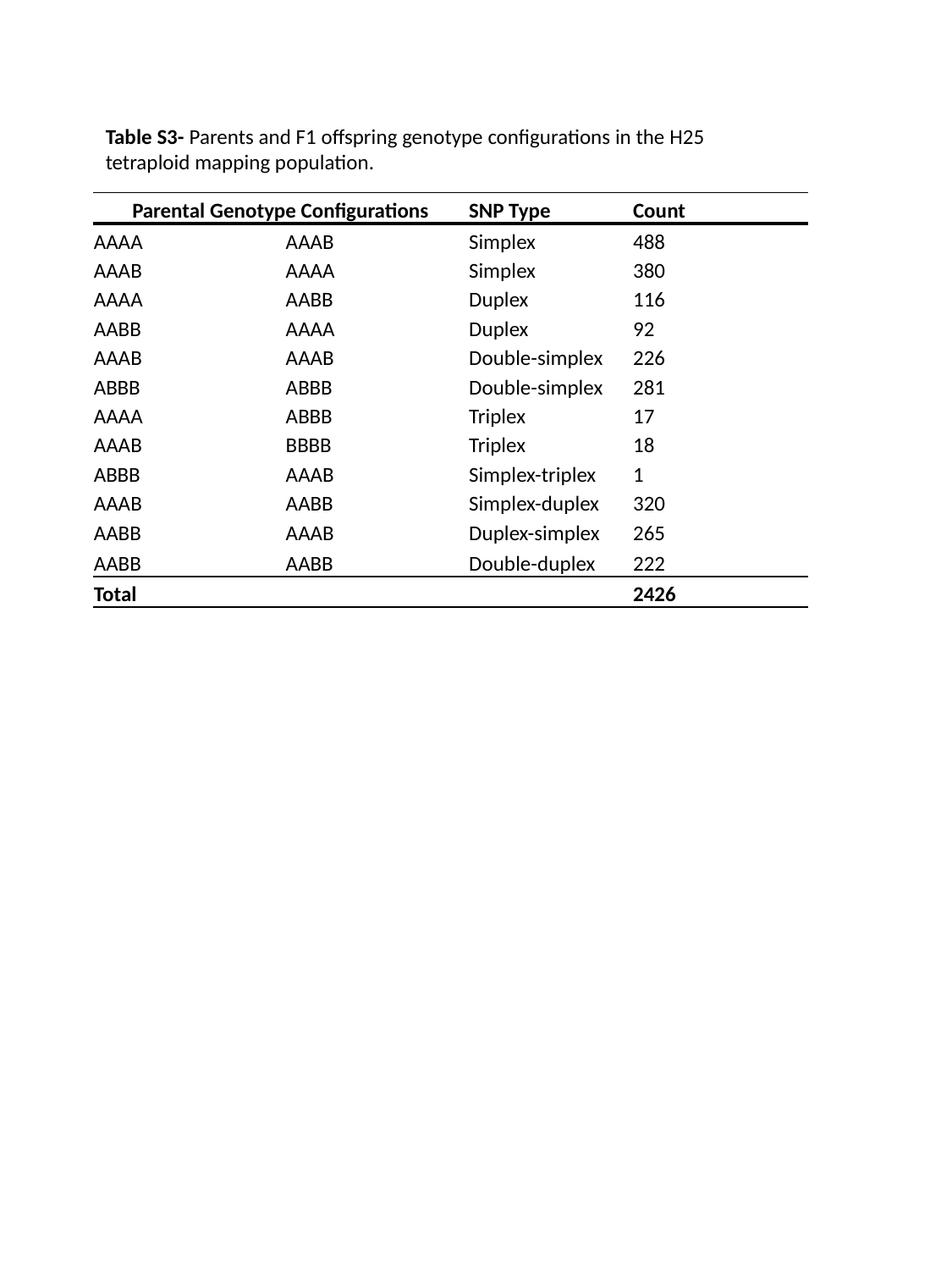

Table S3- Parents and F1 offspring genotype configurations in the H25 tetraploid mapping population.
| Parental Genotype Configurations | | SNP Type | Count |
| --- | --- | --- | --- |
| AAAA | AAAB | Simplex | 488 |
| AAAB | AAAA | Simplex | 380 |
| AAAA | AABB | Duplex | 116 |
| AABB | AAAA | Duplex | 92 |
| AAAB | AAAB | Double-simplex | 226 |
| ABBB | ABBB | Double-simplex | 281 |
| AAAA | ABBB | Triplex | 17 |
| AAAB | BBBB | Triplex | 18 |
| ABBB | AAAB | Simplex-triplex | 1 |
| AAAB | AABB | Simplex-duplex | 320 |
| AABB | AAAB | Duplex-simplex | 265 |
| AABB | AABB | Double-duplex | 222 |
| Total | | | 2426 |

## Slide 6
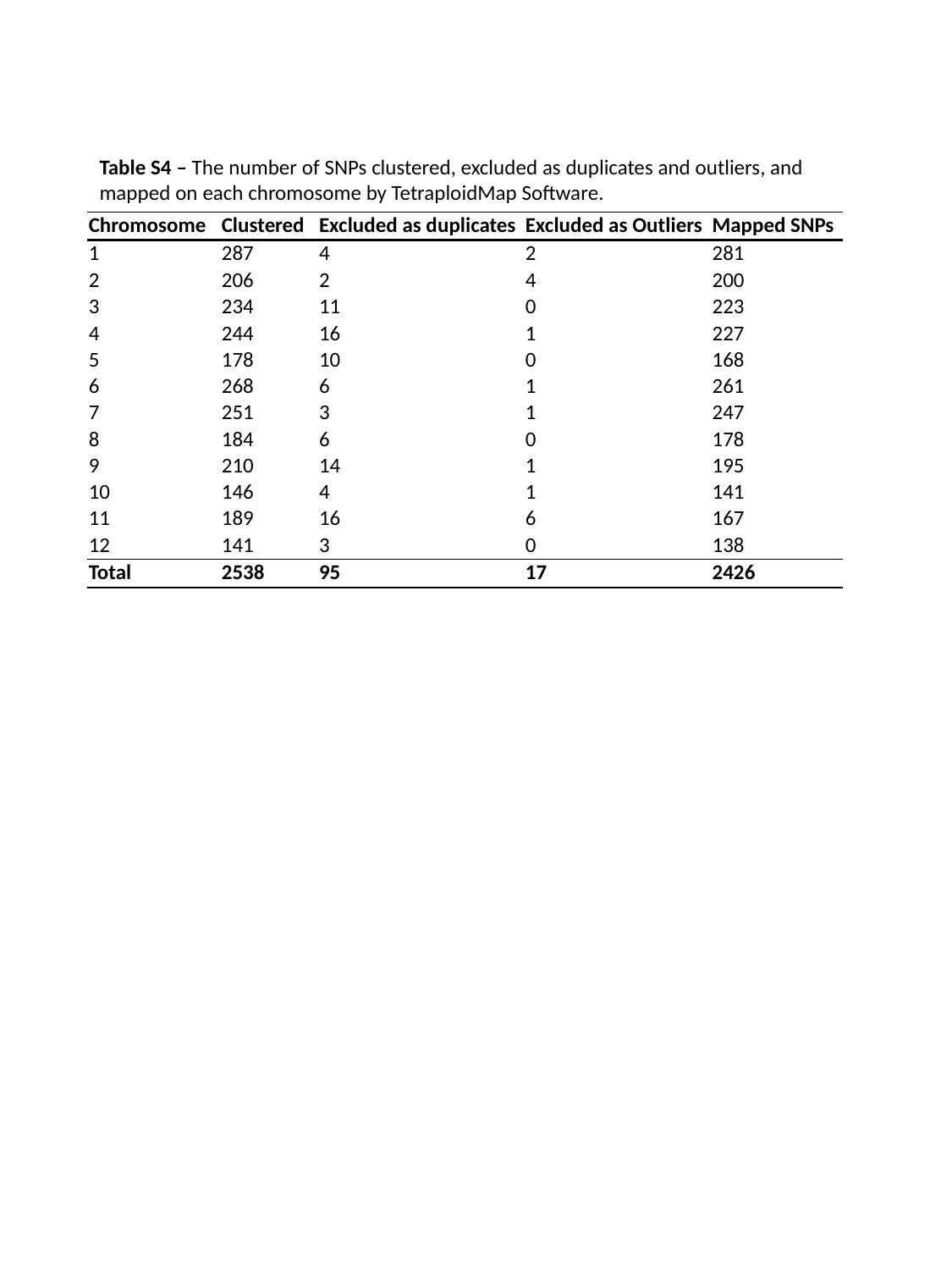

Table S4 – The number of SNPs clustered, excluded as duplicates and outliers, and mapped on each chromosome by TetraploidMap Software.
| Chromosome | Clustered | Excluded as duplicates | Excluded as Outliers | Mapped SNPs |
| --- | --- | --- | --- | --- |
| 1 | 287 | 4 | 2 | 281 |
| 2 | 206 | 2 | 4 | 200 |
| 3 | 234 | 11 | 0 | 223 |
| 4 | 244 | 16 | 1 | 227 |
| 5 | 178 | 10 | 0 | 168 |
| 6 | 268 | 6 | 1 | 261 |
| 7 | 251 | 3 | 1 | 247 |
| 8 | 184 | 6 | 0 | 178 |
| 9 | 210 | 14 | 1 | 195 |
| 10 | 146 | 4 | 1 | 141 |
| 11 | 189 | 16 | 6 | 167 |
| 12 | 141 | 3 | 0 | 138 |
| Total | 2538 | 95 | 17 | 2426 |
